# Effect of COVID-19 pandemic on orofacial and respiratory infections in ear, nose, and throat and oral and maxillofacial surgery emergency departments: a retrospective study of 7900 patients

**DOI:** 10.1007/s00405-021-07107-7

**Published:** 2021-10-01

**Authors:** Aleksi Haapanen, Johanna Uittamo, Jussi Furuholm, Antti Mäkitie, Johanna Snäll

**Affiliations:** 1 Department of Oral and Maxillofacial Diseases, University of Helsinki and Helsinki University Hospital, 00029 HUH Helsinki, Finland; 2 Department of Otorhinolaryngology – Head and Neck Surgery, University of Helsinki and Helsinki University Hospital, Helsinki, Finland

**Keywords:** Infections, COVID19, OMFS, ENT

## Abstract

**Objectives:**

The study purpose was to evaluate the effects of the COVID-19 pandemic on the rate and disease profile of orofacial and respiratory infections in oral and maxillofacial surgery (OMFS) and ear, nose, and throat (ENT) emergency units.

**Materials and methods:**

Records of patients with orofacial or respiratory infection, or infectious symptoms, diagnosed in the OMFS or ENT Emergency Departments of the Helsinki University Hospital, Helsinki, Finland between 1st March and 30th October 2020 and the corresponding periods in 2018 and 2019 were reviewed. The main outcome variable was the occurrence of studied infections during the evaluated periods. Other study variables were age, gender, residence area, speciality, specific cause for the emergency department visit and admission to ward.

**Results:**

There was a significant 37% decrease in the number of infection patients in 2020 compared to the years 2019 and 2018 (1894 vs. 2929 and 3077, respectively, *p* < .001). A mean decrease of 51% (from 1319 and 1249 patients in 2018 and 2019, respectively, to 592 patients in 2020) was seen in the “Other ENT respiratory infection” category. ENT patients were 51% less likely to be admitted to the ward in 2020 compared to 2019 and 2018 (*p* = .013).

**Conclusion:**

A significant decrease was observed in the volume of emergency department visits for orofacial and respiratory infections during the COVID-19-pandemic in 2020 compared to the non-COVID periods.

**Clinical relevance:**

It seems that social distancing, facial mask wearing, and other infection prevention precautions have changed the accustomed patient profile in orofacial and respiratory infections.

## Introduction

Acute upper respiratory infections, like acute rhinosinusitis and odontogenic infections, are commonly found in patients of all ages. Most of these infections are mild and can therefore be treated without hospitalization [1, 2]. They are nevertheless potentially life threatening, since infections in the head and neck may affect the airways by spreading deeper into the orofacial and neck region.

The COVID-19 infection is transmitted mainly by aerosols in the upper aerodigestive track, which can infect by direct contamination or by transmission of respiratory droplets or by airborne transmission [3]. COVID-19 has changed practices in various clinical services [4]. Patient profiles have changed in several surgical fields [5, 6]. The pandemic period has also notably influenced ear, nose and throat (ENT) emergencies, and Stansfield et al. reported a dramatic reduction in the frequency of epistaxis and tonsillar abscesses in both referred and admission patients [7].

The first COVID-19 infection in Finland was found on January 29th in 2020 and the virus then spread around the country (www.thl.fi). As in many other countries, multiple guidelines and instructions were implemented in Finland to avoid an outbreak of COVID infections. For example, the use of masks and better hand hygiene in addition to social distance were instructed (www.thl.fi).

The COVID-19 pandemic has had an enormous effect around the world, as well as numerous indirect effects on the healthcare system. Our aim was to evaluate the effects on the rate and disease profile of orofacial and respiratory infections in oral and maxillofacial surgery (OMFS) and ear, nose, and throat (ENT) emergency units. We hypothesized that the instructions, such as increased hand hygiene and social restrictions, caused by COVID-19 outbreak led to a decrease in non-COVID-19-related orofacial and respiratory infections.

## Materials and methods

### Study design

A retrospective study was performed to clarify the effect of COVID-19 restrictions on the occurrence and severity of different types of orofacial and respiratory infections. Records of patients with orofacial or respiratory infection or infectious symptoms diagnosed in the Oral and Maxillofacial Surgery (OMFS) or ENT Emergency Departments of the Helsinki University Hospital (HUS), Helsinki, Finland between 1st March and 30th October 2020 and corresponding periods in 2018 and 2019 were reviewed. HUS is a tertiary care academic teaching hospital and its departments have a catchment area of approximately 1.6 million inhabitants.

### Inclusion and exclusion criteria

Data retrieval was based on patient ICD-10 diagnostic codes presented in Table 1, of which infection and/or infection suspicion of an acute infectious disease in the orofacial and respiratory system was determined as inclusion criteria. Altogether, 276 ICD-10 diagnoses were included, excluding patients with otologic diagnoses to avoid the possible confounding factor of external otitis.

**Table 1:** ICD-10 diagnose groups evaluated for the study

A30-A49	Other bacterial diseases
A65-A69	Other spirochetal diseases
B00-B09	Viral infections characterized by skin and mucous membrane lesions
B25-B34	Other viral diseases
B35-B49	Mycoses
J01-J06	Acute upper respiratory infections
J9-J18	Influenza and pneumonia
J20-J22	Other acute lower respiratory infections
J30-J39	Other diseases of upper respiratory tract
J40-J47	Chronic lower respiratory diseases
J80-J84	Other respiratory diseases principally affecting the interstitium
J90-J94	Other diseases of the pleura
J95	Other diseases of the respiratory system
J96-J99	Intraoperative and postprocedural complications and disorders of respiratory system, not elsewhere classified
K00-K14	Diseases of oral cavity and salivary glands
L00-L08	Infections of the skin and subcutaneous tissue
L10-L14	Bullous disorders
L20-L30	Dermatitis and eczema
L40-L45	Papulosquamous disorders
L49-L54	Urticaria and erythema
L60-L75	Disorders of skin appendages
L91	Hypertrophic disorders of skin
L98	Other disorders of skin and subcutaneous tissue, not elsewhere classified
R00-R09	Symptoms and signs involving the circulatory and respiratory systems
R10-R19	Symptoms and signs involving the digestive system and abdomen
R20-R23	Symptoms and signs involving the skin and subcutaneous tissue
R50-R69	General symptoms and signs

### Study variables

The main outcome variable was the occurrence of the studied infections during the evaluated periods. Other study variables were age, gender, residence area (metropolitan/rural), specialty (OMFS/ENT), specific cause for the emergency department visit, and admission to the ward (yes/no). Specific causes for visit are categorized in Table 2.

**Table 2:** Cause for emergency department visit during the pandemic period compared to non-pandemic periods

	2018		2019		2020		% change compared to 2018 and 2019	*p* < .001
	*n*	% of *n*	*n*	% of *n*	*n*	% of *n*		
Local dental infection	224_a_	7.3	199_a_	6.8	142_a_	7.5	− 33	
Salivary gland	160_a_	5.2	161_a_	5.5	114_a_	6.0	− 29	
Oral mucosa	61_a_	2.0	28_b_	1.0	26_a,b_	1.4	− 42	
Swelling/abscess in the facial region (excl. peritonsillar abscess)	183_a_	5.9	144_a_	4.9	121_a_	6.4	− 26	
Peritonsillar abscess	703_a_	22.8	717_a_	24.5	555_b_	29.3	− 22	
Other ENT respiratory infection	1319_a_	42.9	1249_a_	42.6	624_a,b_	32.9	− 51	
Dermal or subdermal (incl.lymphadenopathy)	248_a_	8.1	228_a_	7.8	162_a_	8.6	− 32	
Pain/fever/malaise	112_a_	3.6	120_a,b_	4.1	102_b_	5.4	− 12	
Dysphagia/dyspnea/cough	67_a_	2.2	83_a_	2.8	48_a_	2.5	− 36	

*ENT* ear, nose and throat

a, b Different subscript letters denote subsets of categories whose column properties differ significantly from each other in post hoc analysis

### Statistical analysis

Statistical software package IBM SPSS for Macintosh (version 27.0, IBM Corp., Armonk, NY, USA) was used for data analyses. Between the study years, continuous variables were compared with one-way analysis of variance with Bonferroni test as post hoc and categorical variables with Pearson’s *χ*^2^ with *z* test as post hoc. Effect sizes were evaluated with *η*^2^ for continuous and Cramér’s V for categorical variables. *p*-values below 0.05 were considered to be statistically significant throughout the study.

## Results

**Fig. 1:**
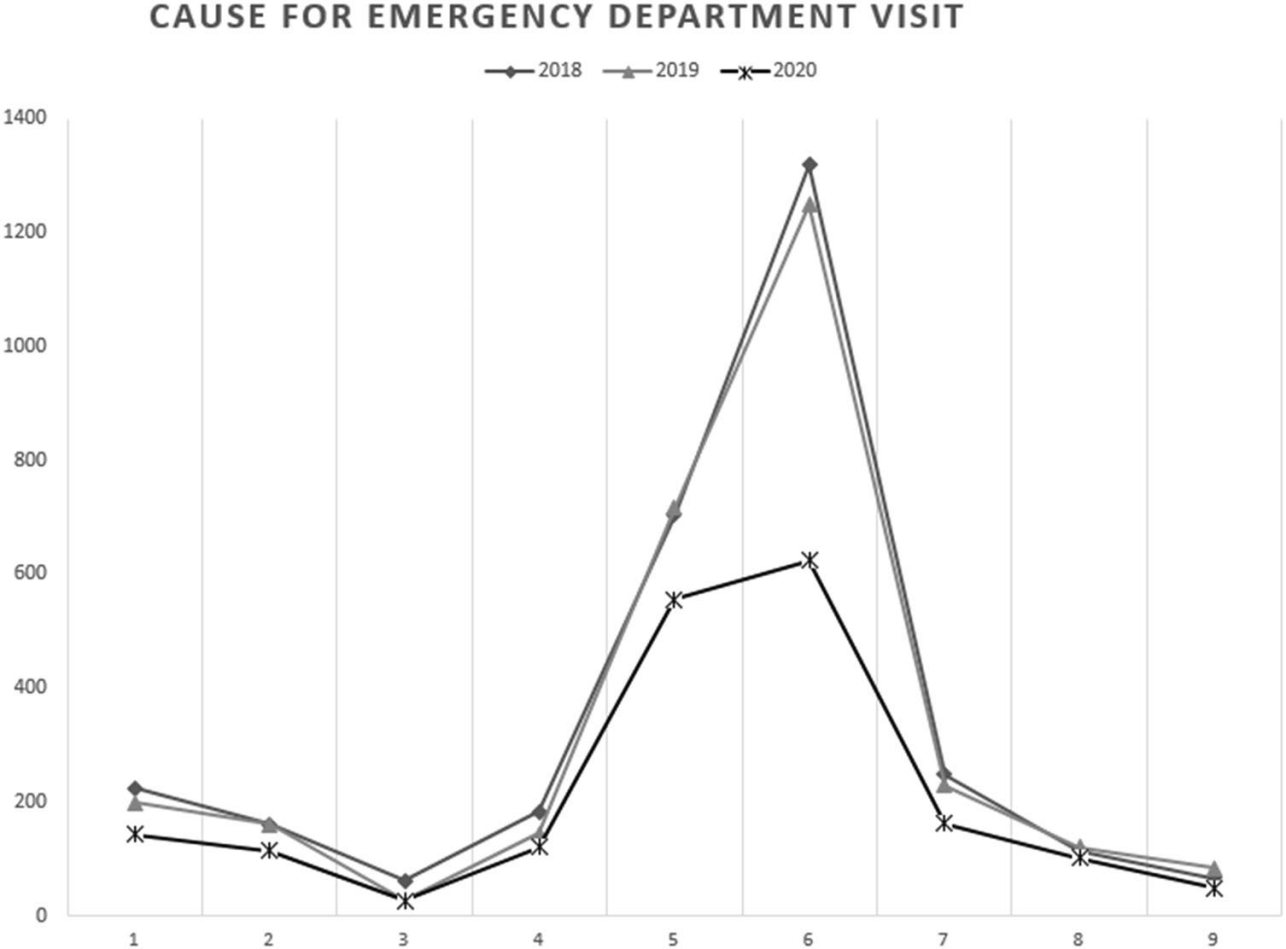
**1** Local dental infection. **2** Salivary gland. **3** Oral mucosa. **4** Swelling/abscess in the facial region (excl. peritonsillar abscess). **5** Peritonsillar abscess. **6** Other ENT respiratory infection. **7** Dermal or subdermal infection (incl. lymphadenopathy). **8** Pain/fever/malaise. **9** Dysphagia/dyspnea/cough

Altogether, 7900 patients (3077 in 2018, 2929 in 2019, and 1894 in 2020) were included in the analysis. There was a significant mean decrease of 37% in the infection patients in 2020 compared to the previous years of 2019 and 2018 (1894 vs. 2929 and 3077, respectively, *p* < .001, *η*^2^ = 0.789. Table 3; Fig. 1). ENT patients were 51% less likely to be admitted to the ward in 2020 compared to 2019 and 2018 (*p* = .013) and patients from rural areas were less likely to visit OMFS or ENT emergency departments during the study period in 2020 compared to 2019 and 2018 (26.8% vs. 29.0% and 30.3%, *p* = .031, *V* = 0.030). Descriptive statistics are found in Table 3.

**Table 3:** Emergency visits for orofacial and respiratory infections during COVID-19 pandemic in OMFS and ENT departments

	2018		2019		2020		% change compared to 2018 and 2019	*p* value
	*n*	%	*n*	%	*n*	%		
Total number of patients (*n* = 7900)	3077	38.9	2929	37.1	1894	24.0	− 37	< .001
Age								
Range	0–100		0–94		1–96			
Mean (median)	37.7 (34)		37.5 (34)		38.2 (34)			.389
Gender								
Male	1458	47.4	1345	45.9	912	48.2	− 35	.278
Female	1619	52.6	1584	54.1	982	51.8	− 39	
Residence area								
Metropolitan area	2144	69.7	2079	71.0	1386	73.2	− 34	.031
Rural	933	30.3	850	29.0	508	26.8	− 43	
Emergency department								
OMFS	423	13.7	361	12.3	276	14.6	− 30	.065
ENT	2654	86.3	2568	87.7	1618	85.4	− 38	
Admitted to ward from the emergency department by specialty								
OMFS	423		361		276		− 30	.073
Yes	73	17.3	85	23.5	51	18.5	− 35	
No	350	82.7	276	76.5	225	81.5	− 28	
ENT	2654		2568		1618		− 38	.013
Yes	334	12.6	333	13.0	163	10.1	− 51	
No	2320	87.4	2235	87.0	1455	89.9	− 36	

*ENT* ear, nose and throat, *OMFS* oral and maxillofacial surgery

Significant changes between the study years were detected in the distribution of reasons for the emergency department visit (*p* < .001, *P* = .073, Table 2). The most drastic mean decrease of 51% (from 1319 and 1249 patients in 2018 and 2019, respectively, to 592 patients in 2020) was seen in the “Other ENT respiratory infection” category. This category included other respiratory infections treated by these specialities excluding peritonsillar abcesses.

## Discussion

**Fig. 2:**
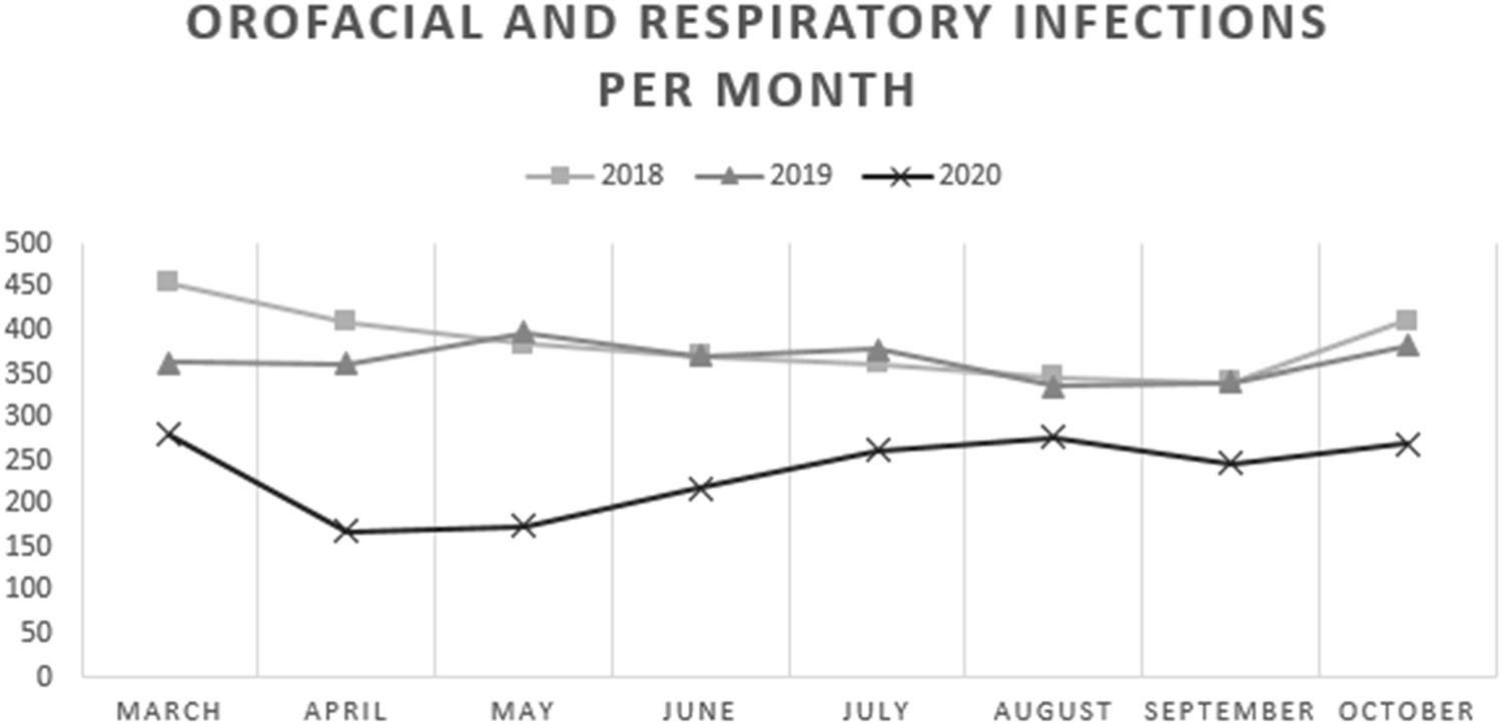
Monthly patient cases of orofacial and respiratory infections in 2018, 2019, and 2020

The aim of this study was to evaluate the effect of COVID-19 restrictions on the rate and disease profile of orofacial and respiratory infections. Our hypothesis was confirmed, as the COVID-19 pandemic led to a significant 37% reduction in the total number of patients with orofacial and respiratory infections in 2020 compared to previous years (*p* < .001). Similar findings have been reported in France and the UK [8, 9]. In our results, the reduction was dramatic in the early months of the pandemic and although it recovered slightly, it did not normalize during the study period (Fig. 2). One possible reason for this finding is that the decrease in elective ENT surgery and public dental care due to the pandemic has led to a reduction in postoperative infection complications effecting the total number of patients attending OMFS or ENT emergency departments. There are however other more plausible explanations for this significant decrease.

Several studies have shown a reduction in ENT patients seeking medical care during the COVID-19 pandemic [7, 10, 11]. Stansfield et al. reported a dramatic reduction in tonsillar abscesses in both referred and admission patients [7]. They found that the total number of tonsillar infection referrals reduced from 136 in 2019 to 29 in 2020 and the admissions to the ward decreased drastically from 112 in 2019 to 2 in 2020. Although these findings were from only a limited series, the findings are in line with our results. The most drastic reduction in patient volume was in patients with respiratory infections and peritonsillar abscesses. One main reason for this decrease could be social distancing and the use of facial masks, which also reduces the spread of infection causing pathogens.

The most severe infections in the facial area require admission to ward and these infections often require surgical treatment in combination with antibiotic medication [12, 13]. Deep neck infections originate most often from an odontogenic cause [12, 14]. Other typical infection sources leading to hospitalization include peritonsillar abscesses, salivary gland infections, iatrogenic causes, intravenous drug use, and infected cysts or tumors [14]. In our data, there was no statistically significant decrease in odontogenic infections during the study period (*p* = .065). Ogle et al. showed that the most common cause for odontogenic infections is periapical infection of the tooth caused by decay, which is a long process and not dependent on the incidence of transmittable pathogens [15]. However, despite the lack of statistically significant differences in our data between the study years, there was a reduction in every diagnosis category in 2020 compared to previous years. This shows that the pandemic has had a wide impact on emergency patients treated in the OMFS and ENT departments.

We found a 51% reduction in ENT patients admitted to the ward. This might be explained by the 21% deduction in peritonsillar abscesses. According to our data this disease group comprises approximately 23–29% of the ENT emergency patients. Severe ENT infections are typically caused by transmittable pathogens such as beta-hemolytic streptococci and *Staphylococcus aureus* [16]. On the other hand, several ENT infections also develop as a sequela of viral infections. According to the present findings, typical ENT emergency infections could be partially prevented by the same preventive measures as the ones used due to the COVID-19-pandemic.

Pandemic drug research, vaccines and other innovations developed during the COVID-19 pandemic, as well as the novel research data on infection spread, will certainly improve diagnostics and treatment of orofacial and ENT respiratory infections. Focus should now be on the spread and prevention of non-COVID-19-related infectious diseases. Further research on the effectiveness of different treatments and practices may yield novel health-promoting findings in tandem with confronting the challenge of the pandemic.

This study has some limitations. The patients presented with a heterogenic group of diseases and symptoms. No detailed analysis was performed for each diagnostic code. Data retrieval was based on the diagnosis of the emergency visit, thus, not on the possible specified diagnosis confirmed later during further care. Therefore, it is possible that some infections are inaccurately or incorrectly registered in the data. The data include only patients who visited the emergency department, and it is likely that in 2020 some of the patients were treated in other departments with the help of specialist consultations to avoid unnecessary patient transition between different units.

## Conclusions

A significant reduction was found in emergency department visits due to orofacial and respiratory infections during the COVID19-pandemic in 2020. These data are useful for the decision making during future health crisis. The findings raise the question of whether some of these preventive measures, such as facial mask policies, should also be used during non-pandemic times to prevent transmission of infectious diseases and further infection complications. Further studies are needed to clarify which measures could be the most cost-effective, taking into account normal social practices.
